# Medical News

**Published:** 1856-08

**Authors:** 


### THE MEDICAL EXAMINER.

### PHILADELPHIA, AUGUST, 1856.

### MEDICAL NEWS.

CHANGES IN MEDICAL SCHOOLS.—Dr. J. M. Allen has resigned the Chair of Anatomy in the Medical Department of the Pennsylvania College.

Dr. T. G. Richardson of Louisville, Ky., has been elected to fill the vacancy, and we are glad to learn accepts the appointment. Dr. Richardson has been long connected with the Louisville Schools, and is well known as a successful teacher of Anatomy; he is also author of a work on that subject.

Dr. Austin Flint has resigned the Chair of Medicine, which he had occupied with so much ability for four years in the University of Louisville, and has accepted the professorship of Clinical Medicine and Pathology in the University of Buffalo, of which he was one of the founders, and for several years one of the chief ornaments. The Chair vacated by Dr. Flint has been filled by the transfer of Professor Rogers; and the Chair of Materia Medica is now occupied by Dr. Robert Breckenridge. Dr. T. G. Richardson has resigned the Demonstratorship of Anatomy, and Dr. Archie Cook, late Professor of Anatomy in the Kentucky School of Medicine, has been called to the vacant post.

The Kentucky School of Medicine has lost, by resignation, Dr. Lawson, Dr. Breckenridge and Dr. Cook, and gained Dr. T. S. Bell and Dr. —— Marshall, of this city. Dr. Ackley has resigned the Chair of Surgery in the Cleveland Medical College. Dr. Edward M. Moore, of Rochester, has been appointed Professor of Surgical Anatomy and Surgical Pathology in the University of Buffalo.

Dr. T. R. Jennings has been elected to the Chair of Anatomy in the University of Nashville, vacated by the death of Dr. R. M. Porter.

Dr. J. B. Biddle has been elected Physician to the Girard College, in the place of Dr. Sargent, resigned.

---

*Examining Board.*—A Board of Medical Officers for the examination of candidates for appointment in the Medical Staff of the Army will convene at Newport Barracks (Ky.) on the first of August next.

Dr. H. A. Ramsay, of Georgia, whose statistics on midwifery were the cause of no little trouble and ill feeling at the meeting of the Am. Med. Association in 1851, and who afterwards started a medical journal called "The Blister and Critic," has lately been arrested, by orders from Washington, on the charge of fabricating testimony in support of false pension claims. He procured $5,000 bail, which was forfeited by his absconding immediately. *Boston Med. and Surg. Journ.*

---

The State Medical Society of Virginia, at its late annual meeting, were compelled to adjourn for a want of a quorum.—*Ibid.*

---

Health of our City.—Our city has so long been in a state of almost unprecedented health, that we have for some time thought it unnecessary to do more than publish, in each number, the list of mortality, a reference to which would satisfy all interested. Within the past month, however, the list of mortality has increased considerably, giving rise, in some degree, to exaggerated reports abroad, with regard to the existence of yellow fever here. It, therefore, behooves us to state the facts of the case.

Personal reference to the books of the Board of Health, show clearly that the health of the adult population of New Orleans is good, and that the mortality list has been increased by the appearance of measles, scarlatina, hooping cough, and cholera infantum, amongst the little children—to say nothing of the effects of "dentition." The quotation of one fact may prove satisfactory on this point. For the week ending Sunday, the 22nd June, there were 35 burials in St. Patrick's Cemetery, and of these 29 were little children. Another cause of the increased mortality is to be found in the great number of deaths from phthisis during the past six months. It is well known that persons afflicted with this disease flock here from every part of the country, and as the summer opens they die off. As to rumors of yellow fever, they are totally unfounded. Up to the present date, (June 25th,) there has not been a single well authenticated case. We have made every endeavor to search out even the one or two reported cases, but could not find them. The fact is, the Charity Hospital is the unerring index to the sanitary condition of New Orleans. When yellow fever begins, it is amongst the class of persons who patronize that institution, and there we are sure to find as early cases as occur in the city. Up to this time there has not been a case of the disease, and the indications are, so far, favorable, as all the fevers there are of a mild and manageable type.—*N. O. Med. News.*

A colored woman in Arkansas recently gave birth to four little darkies—three girls and one boy. The whole lot weighed twenty-eight pounds. At the last accounts the mother was *better* than "could be expected," and the little woolly heads were all as lively as crickets. The given names of the girls are Mississippi, Ouachita and Red River, while the boy is called by the go-ahead name of Railroad. The wife of Mr. Washington French, in Mississippi, has just presented him with two boys and two girls at one birth. Mr. French was forty-five years old when he married, and has been married one year. Though commencing late, he is likely to be surrounded by a large family before old age overtakes him. Thus it will be seen that our "glorious country" is still in advance of the rest of the world, including France, in the baby producing business—to say nothing about great names for "little responsibilities"—as well as in everything else.—*N. O. Med. News.*

---

We copy the following note, as the same error exists in the American edition:

[To the Editor of the Medical Times and Gazette.]

Sir,—Will you have the goodness to allow me space in your Journal to correct an error which some friends have pointed out in one of my works. At p. 86 of "The Diseases of Women," the dose of strychnine is stated to be "from one-tenth or one-fourth of a grain to a grain, three times a day." Recent revelations were not needed to prove that the latter dose would be a poisonous one, nor can I explain how such a mistake happened. I have always commenced with one-sixteenth of a grain, and have never exceeded one-twelfth or one-tenth of a grain. However the error may have happened, I feel it a duty to myself to point it out, and to express my regret for it.

F. Churchill.

15 *Stephen's-green, Dublin, June* 13, 1856.

---

The case of Palmer, although concluded some time ago, still occupies a large space in the English Journals. The medical testimony, we regret to say, was not, on the whole, such as was calculated to elevate the profession in the mind of the public. "There is no doubt whatever," says the Medical Times and Gazette, "but that the estimation in which medical evidence is held by society has, to use the lightest expression, not been improved by the testimony given at the trial." The following extract from the Pharmaceutical Journal, while it shows the difficulty with which the case was environed, contains much matter for reflection to those who may be called upon to testify under similar circumstances:

"PALMER'S TRIAL is the subject which has of late chiefly engaged the public attention. A more important case to the toxicologist has not occurred for many years, and it will furnish materials for discussion in the medical journals for some time to come. We have not given any report of the case in this number, as the trial had not concluded in time even if our space had admitted of it; but we intend to place on record next month an abstract of that portion of the evidence which is interesting in a scientific point of view, and which may be useful for future reference. A complete report, comprising the circumstantial details of the slippery proceedings of a gang of blacklegs would be out of place in this Journal, and could serve no good purpose anywhere, unless as a warning and as furnishing materials for humiliating reflection on the depravity of human nature. But it is sufficiently humiliating to read some of the professional evidence, and to see science and truth decked in fancy dresses, and so disguised as to be available on either side, according to the weight of the retainer which turns the scale. It may be difficult to divest the mind of prejudice, and to avoid leaning towards the side from which the instructions are received. In the legal profession the ends of justice are balanced between the advocates, each being at liberty *per fas et nefas*, to get a verdict if he can. The more ugly the case (within certain limits) the greater the renown acquired by a victory. This license, however, may be indulged in to a disgraceful extent, and if it should transpire that such has been the case, even a legal advocate may outwit himself, and acquire notoriety at the expense of character. But there is a wide difference between the functions of an advocate and those of a witness. No license is granted to the witness to deviate from the strict meaning of the words 'the truth, the whole truth, and nothing but the truth;' and there is no class of witnesses in whom an adherence to this rule is more important than it is in those who are called upon to give evidence on abstruse matters of science, not generally understood by the public, and therefore requiring to be stated in the clearest manner, divested as much as possible from technicalities. A scientific witness, with his head full of hard words and abstract theories, may mystify judge and jury, and defeat the ends of justice by a pedantic display of his knowledge and an ingenious suggestion of possibilities which may or may not be foreign to the question at issue. Those whom it is his duty to inform with regard to matters of fact, being unable to follow him in his theoretical flights, and not understanding the drift of the argument, are likely to attribute importance to that which may have been suggested merely to lead them on the wrong scent. We have on several occasions pointed out this abuse of power, on trials in which chemical witnesses were arrayed against each other, and the court confused with conflicting statements, irrelevant or irreconcilable. These remarks do not apply generally to the witnesses in Palmer's trial. Some of those to whom they are applicable have already been commented upon, and the scientific evidence will be further scrutinized by the profession. As this case will be cited on future occasions, it is important that the facts should be sifted by impartial authorities, and the truth satisfactorily established. The prisoner is not the only party concerned, but future verdicts will be affected by the decision on some of the questions at issue. It was truly remarked by the Attorney-General, that if it be taken for granted that strychnia had not been administered because it had not been found in the body, this doctrine would enable many a guilty man to escape, who, by administering the smallest quantity necessary to destroy life, might prevent its detection in the stomach. It was admitted by witnesses on both sides that the portion of the poison which kills is absorbed in the blood, and it is, therefore, only the excess over and above the fatal dose which would be found in the stomach. It must be obvious to any impartial person who considers the evidence respecting the *post-mortem* examination, that the mode in which it was conducted, and the condition in which the portions of the body were sent for analysis, were most unfavorable for the elucidation of the truth. The stomach, instead of being carefully removed with its contents, was cut from one end to the other, the contents taken out and said to have been afterwards thrown into a jar containing intestines and other portions and contents of the body. Whether the whole of the contents of the stomach, or only a portion or none at all, were thrown into the jar, may admit of some doubt, as the statements on the subject are rather confused. Moreover, the party interested in the suppression of evidence was present, and was rather officious in his interference with the proceedings; he was not then an object of definite suspicion, and was not watched so closely as he would have been if this had been the case. Under these circumstances, with this confused mass of materials, Dr. Taylor commenced his investigation. Although strychnia had been mentioned to him among other poisons which the prisoner had in his possession, the circumstantial evidence forming the principal ground of suspicion with regard to that poison had not then transpired. He found the tissues of the body saturated with antimony. This unusual circumstance led him to pursue his experiments in that direction, and he gave evidence accordingly at the inquest. After he had given his evidence, other witnesses were called, who described the symptoms under which the deceased had died, with other facts relating to the behaviour of the prisoner, and the administration of medicine. Upon this the conviction on his mind was irresistible that strychnia must have been the cause of death, and he gave his opinion to that effect, as the result of the evidence which he had heard. He afterwards renewed his investigation with the remaining portion of materials with reference to strychnia, and experimented on several animals to obtain confirmatory results. But the process for detecting antimony would destroy the materials experimented on, so far as relates to the chance of detecting an organic poison, such as strychnia. Those portions, therefore, which had been so dealt with were no longer available; and if they had contained any amount of strychnia, the evidence was lost. In the materials that remained, no strychnia was found; but this proves nothing. It neither proves that no strychnia was administered, nor does it imply neglect or incompetence in the chemical examination.

It would appear from the statements of some of the witnesses that a revolution had taken place in the science of Chemistry in regard to the detection of strychnia. It was the generally-received opinion that the detection of vegetable poisons of this class was attended with considerable difficulty, when mixed with the contents of the stomach and other matters existing in the body. The tests for strychnia, when pure, are as delicate as those for arsenic or any other poison, but it has always been understood, and is stated in all works on the subject, that the difficulty of isolation from extraneous matters greatly perplexes the Chemist, and may lead to negative results even when the poison is known to be present in notable quantity. We are now told by some of the witnesses in this case, that however minute the quantity, however contaminated with the tissues or contents of the body, they could have detected it. We are told that after a lapse of weeks, or even months, the poison may still be found in the body; that it exists in the blood and in the tissues; that the failure of detection, even if less than a grain had been administered, could only arise from some defect in the process. One of the witnesses for the defence incautiously remarked out of Court that he had no doubt as to the cause of death, but 'Dr. Taylor had not gone the right way to work to find the poison;' and, on cross examination, the remark was brought home to him in Court. Another witness on the same side was 'proving so much,' that, we are told, the prisoner became alarmed, and requested that he might be taken down, as he was damaging the case. No less impolitic was the endeavor to account for the symptoms by wild theories about mental excitement on winning a race, apoplexy, epilepsy, bilious disorder, syphilis, convulsions with tetanic complications, &c. Lord Campbell, in summing up, said, 'It is in my opinion indispensable to the administration of justice, that a witness should not be turned into an advocate, nor an advocate into a witness. You must say, gentlemen, whether some of those who were called for the prisoner belonged to the category I have described—that of a witness becoming an advocate.' The most experienced professional witnesses, men whose opinion has the stamp of authority, said, without hesitation, that they were acquainted with no form of natural disease to which the symptoms described could be attributed, and that these symptoms were precisely those which follow the administration of strychnia.

Fortunately for the ends of justice, the chain of evidence was sufficiently conclusive without the confirmation of the presence of strychnia in the body. The prisoner's circumstances were desperate—instant ruin stared him in the face; he was involved with Cook and others in bets, bill transactions, and forgeries; there seemed no escape except by poisoning somebody; he was officious in giving Cook medicine, which was in several cases followed by vomiting; he bought strychnia twice, administered two doses of pills, which were followed by symptoms resembling nothing but the effects of strychnia. The breath had scarcely left his friend's body when he was detected searching his pockets. The betting-book vanished—money which Cook had the day before, also vanished. The prisoner who had no money the day before, was suddenly in funds. Again, his conduct at the inquest—the bribe of £10 offered to the postboy if he would upset the fly with the jar of materials for analysis—the tampering with the Coroner, and interception of letters at the Post-office—the detection of antimony in the body, and numerous minor incidents, completed the chain of evidence sufficiently to remove all doubt as to the guilt of the prisoner.

There never was a case more ably and carefully got up, both on the part of the Crown and on that of the defence. The trial occupied twelve days, and no circumstance was overlooked or disregarded which could by possibility assist in promoting the ends of justice.—*London Pharm. Journal.*

---

Palmer's trial has had the effect of inciting the profession to the discovery of several new tests for the presence of strychnia. We place two of them on record:—

THE FROG TEST FOR STRYCHNIA.—I have been enabled to detect the 1-2500th part of a grain of the acetate of strychnia. The *young* frog fresh from the pools is the most susceptible to the influence of this extraordinary agent. All young animals are more susceptible than the adult of the same species. The frog is most susceptible of all. It is not less strychnoscopic than galvanoscopic. In proceeding with an inquiry we should begin with the frog, because it is the most detective. We may proceed to use other animals, but these can only detect a larger dose of the poison, and they are in nowise more satisfactory. The phenomena in them are less distinctive even than in the frog. In one case I gave one-sixth of a grain of the acetate of strychnia to a cat. It proved fatal. Some time having elapsed, Mr. Lloyd Bullock prepared the contents of the stomach, and we induced strychnism in three frogs in succession. The dose of poison would scarcely have affected another cat or rabbit. A kitten was killed by one fiftieth part of a grain, and an adult cat by one-thirtieth of a grain of the acetate of strychnia. This would, I should think, not have been detectable by another kitten or cat, as taken from the stomach. But many times less would be detectable and demonstrable by means of the strychnoscopic frog.—*Dr. Marshall Hall in Lancet.*

---

TESTS FOR STRYCHNIA.—So many inconsistencies and inaccuracies have lately appeared in the public papers respecting the discovery of strychnia in the dead body, that I think it right to say that there is not any material with which it can be mixed in the animal body, or process of putrefaction that can in any way interfere with its extraction and recognition. Tartar emetic, common salt, a little nitre, bile, sugar, and a score of other things will destroy its reaction when the tests are performed by those who are not acquainted with the principles of chemistry; but in the hands of the adept such difficulties are instantly overcome.

As to the so-called fallacies of the color-tests for strychnia, these also are fallacies only when the tests are improperly performed; but, to do away with all possible sources of doubt and fallacy from the action of external reagents, I may state that the putting of a little strychnia with sulphuric acid, on a piece of platinum foil, then connecting the foil with the positive pole of a single cell of Grove's or Smee's battery, on touching the acid with the negative pole, terminating in a piece of platinum wire, the violet color so characteristic of strychnia is instantly produced.

This mode of experimenting was suggested by the fact that the color-tests for strychnia are due to the action of nascent oxygen; and so delicate is the galvanic test, that it will discover the presence of the 10,000th of a grain of strychnia; and besides this, its very nature is such as to do away with all possible sources of fallacy.

*Laboratory, London Hospital.* HY. LETHEBY.

—

The defence in the case of Palmer, and the whole of the subsequent proceedings to obtain a reprieve, are founded on the assumption that if "strychnine were administered to Cook in his lifetime, it is now in his body, and can be detected by means that are infallable." A portion of the public appears to me to be led into error by this assumption, the fallacy of which has not been sufficiently explained.

When strychnine, as a poison, is received into the stomach, absorption takes place; the absorption may be more or less rapid, and a portion may or may not remain in the alimentary canal at the period of death. This appears to be admitted. By absorption the poison is received into the blood, it is carried by the bloodvessels to the heart, and propelled, in admixture with the blood, by the heart with almost incredible rapidity to all parts of the body. When it reaches the nervous centres in sufficient quantity, it produces those tetanic and other symptoms which result in death.

The action of many poisons thus received into the blood is extremely rapid and transient, and they cannot remain long in the blood. There are glands, the office of which are, in fact, to separate such substances from the blood, and they are accordingly more or less rapidly separated in the secretions, as the urine, the saliva, or the perspiration, and may be detected there. That strychnine is not an exception to this law is shown by the evidence of Mr. Herapath, who detects it in the urine.

In the case of Cook, who is to affirm that, while, on the one hand, the poison was producing its deadly effects on the nervous system, on the other, it was not being separated into one or more of the secretions? On what ground can it be assumed that at the moment of death one particle remained in the blood, or any organ or structure, to tell its tale?

Two grains of strychnine, if pure, are sufficient to produce its deadly effects. There is believed to be about 25 lbs. of blood in the adult human body; and the proportion in which strychnine continues to be detectable is variously stated by Mr. Herapath and other witnesses at 1-20,000th to 1-50,000th part of the substances operated on. If the whole of the assumed quantity of strychnine were absorbed at once, and were present, equally diffused in the blood, at one instant, it would constitute, on these data, only 1-96,000th part of that fluid. But when foreign agents, as strychnia, are thus received into the blood by absorption, the process is gradual, and as they reach the heart experience has shown they become irregularly diffused ; so that if a part of the body only be examined; that part may contain more or less or none of the poison. The separation of the poison with the secretions is almost as rapid as the absorption. How rash, then, must the assertion be that strychnine must be necessarily detectable in any particular organ, or in the blood of any organ, or in that obtained from any part of the body; or that it must certainly exist in the body after death.

Professor Taylor has been assailed on the ground that the hypothesis of strychnine being decomposed in the body is an invention of his own. Nothing can be more unjust. The hypothesis, apart from its application to poisoning by strychnine, has been maintained by chemists and physiologists of the highest character for many years. It is a very general, although not an exclusive doctrine, that when foreign agents, such as strychnine, produce their effects on the living body, the decomposition of a portion of the agent results. Liebig holds that the vegetable alkaloids (to which class of chemical substances strychnine belongs), when received into the blood, are decomposed by the action of the oxygen of the atmosphere. Although I think it is to be regretted that Professor Taylor relied exclusively on this explanation of the fact, that strychnine might not be detectable after death by its own agency, there is no ground for the accusation that this gentleman invented the hypothesis to serve a purpose.

Thus, there are three prominent physiological circumstances to which, taken separately or conjointly, the fact may be attributed that strychnine was not discovered, and may not be discoverable in the body of Cook, although he was poisoned with that substance. 1. The too general diffusion of the poison over the mass of the solids and fluids of the body. 2. The decomposition of the poison either during its action in the tissues or during its circulation in the blood. 3. The elimination of a portion of the poison into one or more of the secretions. That Cook's system was under the influence of antimony—a substance possessing the greatest activity in promoting several of the secretions—ought not to be overlooked.—*Henry Ancell, formerly Lecturer on Forensic Medicine in Ass. Journ., from Dublin Medical Press.*

---

Civilization and Depopulation.—The Hawaiian nation, which, seventy years ago, was estimated variously at from 200,000 to 400,000, now only counts 72,000, a decrease within that period of at least two-thirds. Vast tracts of land do not harbor a human soul; fertile kalo lands, once under cultivation, are left to the rule of grass and weeds. The island of Kauai, remarkable for the productiveness of the soil, and capable of sustaining a population of at least 100,000, contains only 6,000. It is not to cruel and devastating wars that we have to attribute this unparalleled falling off in so short a time. The wars of Kamehameha I., however energetically they were carried on, cannot in the remotest degree be compared, so far as waste of life is concerned, with those of modern civilized nations. And it is after those wars, moreover, after the blessings of civilization were transferred hither, that the blight falls most mercilessly on this doomed people. The cause of the evil is an internal one, not caused, but increased, by external influences. its investigation resolves itself naturally into these two questions—the scarcity of births, and the frequency of death.—*Polynesian.*

---

Died, July 1st, Dr. R. M. Porter, Professor of Anatomy in the University of Nashville, from the effects of a dissecting wound. Dr. Porter was an excellent teacher, and a most estimable and high minded gentleman, respected by all who knew him.

Died, in London, May 1st, 1856, aged —, Geo. J. Guthrie, well known as one of the most eminent military surgeons of our day.

Died, in London, on the 24th April, 1856, in the 90th year of his age, Henry Clutterbuck, M. D. Dr. C. was for nearly seventy years an active member of the profession, during all which period his life was one of study and continued industry. For fifteen years he edited the *Medical and Chirurgical Review*, (1795-1809,) and was the author of several valuable works. He was three times President of the London Medical Society, and for upwards of sixty years attended its anniversary meeting.

